# Providing information at the initial consultation to patients with low back pain across general practice, chiropractic and physiotherapy – a cross-sectorial study of Danish primary care

**DOI:** 10.1080/02813432.2022.2139465

**Published:** 2022-10-31

**Authors:** Lars Morsø, Jesper Lykkegaard, Merethe Kirstine Andersen, Anders Hansen, Mette Jensen Stochkendahl, Simon Dyrløv Madsen, Berit Schiøttz Christensen

**Affiliations:** a Department of Clinical Research, Research Unit OPEN, University of Southern Denmark, Odense, Denmark; b OPEN – Open Patient data Explorative Network, Odense University Hospital, Odense, Denmark; c Department of Public Health, Research Unit of General Practice, University of Southern Denmark, Odense, Denmark; d Spine Centre of Southern Denmark, Lillebaelt Hospital, Middelfart, Denmark; e Chiropractic Knowledge Hub, Odense, Denmark; f Department of Sports Science and Clinical Biomechanics, Center for Muscle and Joint Health, University of Southern Denmark, Odense, Denmark

**Keywords:** Low back pain, provision of information, primary care, clinical guidelines, prospective survey registration

## Abstract

**Objective:**

Clinical guidelines for managing low back pain (LBP) emphasise patient information, patient education and physical activity as key components. Little is known about who actually receives information. This study investigates to what extent information at the first consultation with general practitioner (GP), chiropractor (DC) and physiotherapist (PT) in Danish primary care is provided to patients with LBP.

**Design and setting:**

This cross-sectorial study was conducted as a prospective survey registration of LBP consultations at the three primary health care professions in Denmark.

**Intervention:**

Clinicians ticked off a paper survey chart during or after consultations with patients who visited the clinic for LBP (Approval number: ID # 11.220).

**Subjects:**

33 GPs, 43 DCs and 61 PTs registered first-time consultations.

**Main outcome measures:**

The primary outcome was provision of information, overall and across care settings.

**Results:**

The overall proportion of patients provided with information was 72%, but this varied among professions (GP, 44%; DC, 76%; and PT, 74%). Provision of information increased to 78% if patients had increased emotional distress or back-related leg pain below the knee. The strongest association with provision of information was having two or three signs of elevated distress (OR 2.58 and 5.05, respectively, *p*= 0.00) or physical disability (OR 2.55, *p*= 0.00).

**Conclusion:**

In more than a quarter of first-time consultations, patient information was not provided. Large variation in providing information was found across the settings. The proportion provided with information increased for sub-populations having elevated distress or back-related leg pain below the knee.

**Key Points**
 Clinical guidelines recommend patient information, patient education and physical activity for managing low back pain (LBP)  • Information is not provided in more than a quarter of first-time consultations in Danish primary care settings that manage these patients.
 • Information increased for the sub-populations having elevated distress and back-related leg pain below the knee.
 • The conducted primary care surveys monitored clinical activity and illustrated variations in provision of information.

## Introduction

Low back pain (LBP) is common globally [1]. In Denmark, the point prevalence estimate is 13.7% [2], and most primary care consultations regarding musculoskeletal conditions are for LBP [3]. The vast majority of patients with LBP are managed in primary care in general practice or at chiropractor and physiotherapy clinics. The yearly Danish treatment cost for LBP is €240 million, and Danes with LBP have 3.3 million more visits at the general practitioner (GP), and 2.3 million more visits at the chiropractor (DC) and physiotherapist (PT), per year than people without LBP [4].

Internationally, clinical guidelines for managing LBP emphasise patient information, patient education and physical activity as key components [5,6]. In the Danish guidelines, patient information and education are recommended, and the guidelines state that initial LBP management should include information on prognosis, warning signs and staying active [6]. Further, the Danish guidelines recommend patient education consisting of dialog-based reassurance with cognitive and behavioural components to patients with low self-efficacy or self-management skills, or at risk of a poor prognosis [5,6].

Several factors may negatively affect the LBP prognosis, including psychosocial and cognitive components [7–9], disability [10] and back-related leg pain [11]. According to guideline recommendations, LBP patients with indications of poor prognosis, elevated distress or leg pain should receive information and/or education as a central part of the initial management, but the clinical reality may differ from guideline recommendations [12]. Previous studies indicate large variations in clinicians’ approaches to LBP [10,13] and suboptimal compliance with guideline recommendations [12]. For example, in 2013, different health care professionals in Danish primary care gave information regarding back symptoms and prognosis in 60% to 80% of LBP consultations [14]. However, little is known about which patients actually receive information and/or patient education or if information and education are given to those intended, especially if patients with a poor prognosis, distress or leg pain are managed as recommended in the guidelines.

This study aims to investigate to what extent information at the first consultation with one of the three professions of GP, DC and PT in Danish primary care is provided to patients with LBP. We investigate patient characteristics associated with provision of information, and we analyse if patients with elevated psychological/cognitive distress or back-related leg pain below the knee are provided information differently than patients without these characteristics.

## Material and methods

### Design

The study is observational and cross-sectorial. We conducted a consecutive survey registration of consecutive LBP consultations in the three main primary health care provider groups (GP, DC and PT) in Denmark. The registration also included the regional secondary care spine centre, but this paper only regards primary care. The Danish health care system is generally tax-funded and offers free and equal access for all Danish citizens to consult a GP and to receive a subsidy that partly covers expenses for DC and PT treatment in primary care. The subsidy for PT treatment requires a GP referral.

In the study, we used a well-tested paper survey chart method [15] developed by Audit Project Odense (APO). We invited all registered GP, DC and PT primary care clinics in the Region of Southern Denmark (covering approx. 1.2 million inhabitants) to participate in the survey. The survey registration was conducted during November and December 2019. Each clinician was asked to tick off the paper survey chart during or after consecutive consultations with adults (>16 years) who visited the clinic for LBP. The registration durations, 2 weeks for DC and PT clinicians and 4 weeks for GP clinicians, were based on the estimated prevalence of patients consulting for LBP.

All the clinicians participated voluntarily and signed a written consent form before study entry. All collected patient data were anonymous, and no written consent was necessary. In accordance with Danish legislation, the authorised legal department at the University of Southern Denmark approved the study (ID # 11.220).

### The survey

The survey chart contained 45 variables for PTs and 47 for GPs and DCs within ten domains (Appendix 1) and started with registration of the consultation number (i.e. number of consultations during current pain episode). The ten domains covered items assessed during the consultation, including pain location and duration, psychosocial risk factors of LBP, initiated treatment, recommendations on medication, and referral. Most questions (40 of 45/47) were identical across professions, but some were adapted to fit the scope of practice of each profession. The items reflected the scientific literature, the clinical guidelines for LBP [6] and common clinical practice as determined by consensus of a development group within the project with representatives from all three professions. This approach was chosen to ensure coverage of common clinical practice not included in the clinical guidelines. Domains were designed with exhaustive answer categories. If a full domain was left unticked, it was coded as missing. Each registration obtained the clinician’s subjective valuations based on the interaction with the patient. To assist the clinicians with interpretation of the items, a definition and description of each item was included in the survey. Prior to distribution of the survey, the survey chart was pilot tested by a sample of GPs, DCs and PTs and revised according to the feedback. A common coding manual was provided to all participants prior to the survey.

Participating clinicians also completed a background questionnaire containing information on age, gender, profession, experience, type of clinic, primary care collaborators and knowledge about and application of regional management procedures [16] and the STarT Back Tool [7].

### Analysis

All first-time consultations with patients experiencing a new episode of LBP were included in the analysis. The primary outcome was provision of information (reflected in item number 24 ‘information on back symptoms including prognosis’), overall and across care settings. Supplementary analyses were conducted for two subgroups of patients with increased risk of disability [17]. (1) Patients with elevated psychological/cognitive distress (defined as experiencing one of three items of impairments or disability, including poor sleep, emotional distress and 2 or more pain sites in addition to LBP; the three variables were significantly correlated, indicating that all three reflected distress), (2) patients with back-related leg pain below the knee. The two sub-populations were collapsed, and differences for receiving information if ‘not in a sub-population’ versus being ‘in a sub-population’ were tested using Kruskal–Wallis. The overall results were displayed as univariate and multivariate analyses adjusted for setting, age, gender, duration of episode, prior episodes, pain location, distress, sick leave, physical disability and pain medication. Odds ratios (ORs) for provision of information at the initial consultation were analysed and modelled using Stata^®^, release 15.0 (StataCorp, College Station, TX). *p* Values of <0.05 were considered statistically significant.

## Results

In total, 33 GPs registered 149 first-time consultations, 43 DCs registered 683 first-time consultations and 61 PTs registered 308 first-time consultations (Table 1). Overall, 53% of the included patients were women, and the median age was 52 years (interquartile range [IQR] 40–64 years). The median symptom duration at the time of consultation was 2 weeks (IQR 0–8 weeks), and 62% of all included patients had had previous episodes of LBP. Across professions, there were significant differences in all these variables, with PTs seeing more women, patients that were older and patients with longer pain duration (Table 1).

**Table 1.** Baseline patient characteristics. Overall and by profession.

	Total	Chiropractors	Physioterapists	General practitioners	*p* Value^a^
Patient numbers, overall and across professions	*N* = 1140	*N* = 683	*N* = 308	*N* = 149	
Gender, female	600 (52.6%)	336 (49.2%)	180 (58.4%)	84 (56.4%)	**0.016**
Age (years)	52 (40–64)	49 (38–61)	56 (45–70)	52 (41–63)	**<0.001**
No. of weeks with symptoms in current course	2 (0–8)	1 (0–3)	5 (2–46)	2 (0–8)	**<0.001**
Previous back pain episodes, several disabling episodes	700 (62.2%)	451 (66.5%)	181 (59.5%)	68 (47.2%)	**<0.001**
Previous treatment in the current episode					
Chiropractor	59 (13.5%)	0 (0.0%)	40 (13.1%)	19 (14.6%)	0.67
GP	394 (40.5%)	163 (24.4%)	231 (75.5%)	0 (0.0%)	**<0.001**
Secondary care Spine centre/Hospital/operation	96 (8.7%)	42 (6.3%)	45 (14.7%)	9 (6.9%)	**<0.001**
MRI	85 (7.7%)	33 (4.9%)	43 (14.1%)	9 (6.9%)	**<0.001**
Physiotherapist	135 (16.9%)	96 (14.4%)	0 (0.0%)	39 (30.0%)	**<0.001**
Other	598 (54.2%)	457 (68.4%)	64 (20.9%)	77 (59.2%)	<0.001
Findings					
Radiating pain below knee	174 (15.7%)	82 (12.3%)	64 (21.1%)	28 (20.0%)	**<0.001**
Widespread pain (2 or more pain sites beside LBP)	260 (23.0%)	128 (18.9%)	99 (32.1%)	33 (22.3%)	**<0.001**
Poor sleep	473 (41.8%)	281 (41.6%)	139 (45.1%)	53 (35.8%)	0.17
Physical disabled	928 (82.0%)	578 (85.5%)	248 (80.5%)	102 (68.9%)	**<0.001**
Emotional disabled	256 (22.6%)	147 (21.7%)	90 (29.2%)	19 (12.8%)	**<0.001**
Sick leave	148 (13.1%)	93 (13.8%)	42 (13.6%)	13 (8.8%)	0.25
Abnorm neurology	99 (8.7%)	50 (7.4%)	28 (9.1%)	21 (14.2%)	**0.028**
Neurological examination conducted	284 (25.1%)	147 (21.7%)	119 (38.8%)	18 (12.2%)	**<0.001**
Clinical nerve root pressure	88 (7.8%)	47 (6.9%)	26 (8.5%)	15 (10.1%)	0.36
Suspected serious pathology	10 (0.9%)	3 (0.4%)	5 (1.6%)	2 (1.4%)	0.15
Initiated treatment					
Counselling and advice in self-management	888 (78.2%)	538 (79.1%)	259 (84.1%)	91 (61.9%)	**<0.001**
Acupuncture/ Dry needling (including blockade for GPs)	64 (5.6%)	53 (7.8%)	8 (2.6%)	3(2.0%)	**0.002**
Instruction in direction-specific exercises	121 (39.3%)	N/A	121 (39.3%)	N/A	
Instruction in exercises/active exercises	524 (46.0%)	298 (43.6%)	199 (64.6%)	27 (18.4%)	**<0.001**
Information; symptoms and prognosis	811 (71.5%)	519 (76.3%)	227 (73.7%)	65 (44.2%)	**<0.001**
Dialog; lifestyle factors	78 (25.3%)	N/A	78 (25.3%)	N/A	
Manuel Therapy	823 (72.5%)	651 (95.7%)	166 (53.9%)	6 (4.1%)	**<0.001**
NSAID	35 (23.8%)	N/A	N/A	35 (23.8%)	
Opioids	13 (8.8%)	N/A	N/A	13 (8.8%)	
Gabapentin/Lyrica/Tricyclic Antidepressives (TCA)	9 (6.1%)	N/A	N/A	9 (6.1%)	
ULRUS (Extended Back examination)	18 (1.8%)	2 (0.3%)	16 (5.2%)	N/A	
Other	17 (1.5%)	1 (0.1%)	1 (0.3%)	15 (10.2%)	**<0.001**
Purpose of the consultation					
The cause to LBP	835 (73.7%)	545 (80.1%)	226 (73.9%)	64 (43.5%)	**<0.001**
Reassurance	578 (51.0%)	344 (50.6%)	167 (54.6%)	67 (45.6%)	0.19
Fulfil patient demands	454 (40.1%)	346 (50.9%)	85 (27.8%)	23 (15.6%)	**<0.001**
Ease pain	930 (82.1%)	654 (96.2%)	210 (68.6%)	66 (44.9%)	**<0.001**
Improve self-management	773 (68.2%)	456 (67.1%)	259 (84.6%)	58 (39.5%)	**<0.001**
Other	10 (0.9%)	4 (0.6%)	0 (0.0%)	6 (4.1%)	**<0.001**

Data are presented as median (IQR) for continuous measures, and *n* (%) for categorical measures.

^a^Between group differences tested using Kruskal–Wallis for non-parametric testing.

Twenty percent of patients who consulted GPs and PTs had back-related pain below the knee, while this was the case for 12% at DCs. Widespread pain was reported in 19–32% of patients and emotional distress in 13–29%. The proportion of patients with poor sleep was above 40%, and overall, 82% of the patients presented physical disability, varying among professions, with 69% at GPs, 81% at PTs and 86% at DCs. However, only 1% were suspected of having serious pathology (Table 1).

The overall proportion of patients provided with information was 72%, but this proportion varied, with 44% at GPs, 76% at DCs and 74% at PTs. If patients were classified into one of the two pre-defined sub-populations of having increased emotional distress or back-related leg pain below the knee, the overall proportion of patients who were provided with information at the first consultation increased to 78% for both sub-populations. Provision of information for the ‘distress sub-population’ increased to 47% at GPs, 84% at DCs and 77% at PTs. For the ‘leg pain sub-population,’ provision of information increased at GPs to 54% and at DCs to 89%, but decreased at PTs to 73%. Compared with patients not belonging to any of the subgroups, patients in either one of the subgroups were more likely to receive information (Table 2).

**Table 2.** Provision of information at first consultation. Proportion of patients who were provided with information on LBP including prognosis and differences across subpopulations.

	Overall population		Sub population 1^a^		Sub population 2^b^		Test for receiving information if or not in a subpopulation^c^
	Yes	No	Yes	No	Yes	No	*p* Value
Overall	811 (71.5%)	324 (28.6%)	514 (77.9%)	146 (22.1%)	135 (77.6%)	39 (22.4%)	0.000
GP	65 (44.2%)	82 (55.8%)	33 (46.7%)	40 (53.3%)	15 (53.6%)	13 (46.4%)	0.441
Chiropractor	519 (76.3%)	161 (23.7%)	328 (84.1%)	62 (15.9%)	73 (89.0%)	9 (11.0%)	0.000
Physiotherapist	227 (73.7%)	81 (26.3%)	151 (77.4%)	44 (22.6%)	47 (73.4%)	17 (26.6%)	0.048

^a^Sub-population 1 is defined as having increased ‘distress’. Distress is defined by having ‘yes’ on one of the following three variables: 2 or more pain sites beside LBP, disturbed sleep, emotional distress.

^b^ Sub-population 2 is defined by having back related ‘pain below knee’.

^c^ Subpopulation 1 and 2 was collapsed, and differences for receiving information across ‘not in a sub-population’ *vs.* being ‘in a sub-population’ was tested using Kruskal–Wallis for non-parametric testing.

Patient characteristics associated with provision of information at the first visit in the overall population are displayed in Table 3. In the univariate analysis, several variables were associated with the provision of information. The strongest association with the provision of information was having two or three signs of elevated distress (OR 2.58 and 5.05, respectively, *p*= 0.00) and physical disability (OR 2.55, *p*= 0.00). GPs were significantly less likely to provide information (OR 0.25, *p*= 0.00).

**Table 3.** Uni- and multivariate analysis displaying the odds ratio for being provided with information at first consultation in the overall population.

*n* = 1047		Overall							
		Uni-variable				Multi-variable			
		OR	95% CI		*p* Value*	OR	95% CI		*p* Value*
Chiropractor		1	(Reference)			1	(Reference)		
Physiotherapist		0.87	0.64	1.18	0.38	0.86	0.60	1.22	0.40
General Practice		0.25	0.17	0.36	**0.00**	0.29	0.18	0.45	**0.00**
Gender	Female	1	(Reference)			1.00	(Reference)		
	Male	1.05	0.81	1.35	0.73	1.23	0.91	1.65	0.17
Age		0.99	0.99	1.00	0.19	0.99	0.98	1.00	0.22
								
Duration of symptoms (number of weeks)		1.00	1.00	1.01	0.55	1.00	1.00	1.01	0.72
Previous episodes	<2 episodes	1	(Reference)			1.00	(Reference)		
	>2 episodes	0.80	0.61	1.05	0.10	0.54	0.39	0.75	**0.00**
Symptoms below knee	No	1.00	(Reference)			1.00	(Reference)		
	Yes	1.44	0.98	2.12	0.06	1.13	0.74	1.74	0.57
Sick leave	No	1.00	(Reference)			1.00	(Reference)		
	Yes	1.92	1.24	2.98	**0.00**	1.40	0.82	2.37	0.21
Distress (having 2 or more pain sites, poor sleep, emotional disability)									
	No sign of distress	1.00	(Reference)			1.00	(Reference)		
	One sign of distress	1.74	1.30	2.33	**0.00**	1.74	1.25	2.41	**0.00**
	Two signs of distress	2.58	1.72	3.85	**0.00**	2.48	1.56	3.94	**0.00**
	Three signs of distress	5.05	2.26	11.30	**0.00**	4.66	1.98	11.00	**0.00**
Physical disability	No	1.00	(Reference)			1.00	(Reference)		
	Yes	2.55	1.87	3.49	**0.00**	2.19	1.54	3.14	**0.00**

* *p* Values ≤0.05 are considered as significant.

In the multivariate analysis, the association of providing information with distress, physical disability and attending a GP consultation was largely retained, whereas patients having >2 previous episodes were negatively associated with the provision of information (OR 0.54, *p*= 0.00). In the multivariate model, association with sick leave was washed out (Table 3).

Across the three professions, the associations for providing information at the first consultation showed considerable variation. For GPs, the multivariate analysis washed out all significant associations from the overall analysis (Table 4). For DCs, the multivariate analysis revealed significant associations with episode (OR 0.31, *p*= 0.00), level of distress (OR 2.30, 3.37 and 4.10, *p*= 0.00–0.03) and physical disability (OR 2.92, *p*= 0.00). Further, the DCs were more likely to give male patients information compared with female patients (OR 1.48, *p*= 0.05) (Table 4). For PTs, the statistically significant associations in the multivariate analysis were having ‘severe’ elevated distress (all three signs) and physical disability (OR 4.33, *p*= 0.03, and 2.13, *p*= 0.02, respectively) (Table 4).

**Table 4.** Multivariate analysis displaying the odds ratio for providing information at first consultation in the total population. Displayed by profession.

		General practitioners (*n* = 109)				Chiropractors (*n* = 645)				Physiotherapists (*n* = 293)			
		Multi-variable				Multi-variable				Multi-variable			
		OR	95% CI		*p* Value*	OR	95% CI		*p* Value*	OR	95% CI		*p* Value*
Gender	Female	1.00	(Reference)			1.00	(Reference)			1.00	(Reference)		
	Male	0.52	0.23	1.22	0.13	1.49	1.00	2.22	**0.05**	1.09	0.61	1.95	0.77
Age		1.00	0.97	1.02	0.80	0.99	0.98	1.01	0.58	0.99	0.98	1.01	0.56
Duration of symptoms		1.00	0.99	1.03	0.50	1.01	1.00	1.01	0.12	0.99	0.99	1.00	0.15
Previous episodes	<2	1.00	(Reference)			1.00	(Reference)			1.00	(Reference)		
	>2	0.77	0.30	1.97	0.58	0.31	0.19	0.50	**0.00**	1.13	0.63	2.01	0.69
Symptoms below knee	No	1.00	(Reference)			1.00	(Reference)			1.00	(Reference)		
	Yes	0.96	0.34	2.70	0.94	1.95	0.91	4.18	0.08	0.70	0.35	1.38	0.30
Sick leave	No	1.00	(Reference)			1.00	(Reference)			1.00	(Reference)		
	Yes	4.94	0.69	35.3	0.11	1.14	0.57	2.24	0.70	1.97	0.68	5.68	0.21
Distress	No sign of distress	1.00	(Reference)			1.00	(Reference)			1.00	(Reference)		
	One sign of distress	0.50	0.19	1.31	0.16	2.30	1.48	3.57	**0.00**	1.61	0.83	3.13	0.16
	Two signs of distress	3.23	0.76	13.72	0.11	3.37	1.75	6.48	**0.00**	1.34	0.60	2.97	0.47
	Three signs of distress	4.37	0.37	51.9	0.24	4.10	1.12	14.97	**0.03**	4.33	1.21	16.68	**0.03**
Physical disability	No	1.00	(Reference)			1.00	(Reference)			1.00	(Reference)		
	Yes	1.10	0.44	2.74	0.85	2.92	1.76	4.85	**0.00**	2.13	1.13	4.03	**0.02**
Medication	No	1.00	(Reference)			NA				NA			
	Yes	1.14	0.50	2.59	0.76	NA				NA			

*significant level *p*<0.05

The secondary analyses included the two sub-populations defined by elevated distress (subgroup 1) or patients having back-related leg pain below the knee (subgroup 2). Analysis showed that information provision increased in both subgroups compared with patients not belonging to a subgroup. In subgroup 1, multivariate analysis showed that GPs registered provision of information at the first consultation significantly less often (OR 0.21, *p*= 0.00), while physical disability was positively associated with information (OR 1.94, *p*= 0.02) (Table 5).

**Table 5.** Multivariate analysis displaying the odds ratio for provision of information at first consultation in the sub-populations with increased distress^a^ and ‘back related pain below knee’.

(*n* = 611)		Subpopulation ‘Increased distress’				Subpopulation ‘Back related pain below knee’			
		Multi-variable				Multi-variable			
		OR	95% CI		*p* Value	OR	95% CI		*p* Value
Profession	DC	1.00	(Reference)			1.00	(Reference)		
	PT	0.72	0.44	1.18	0.19	0.31	0.12	0.84	**0.02**
	GP	0.21	0.11	0.40	**0.00**	0.17	0.05	0.51	**0.00**
Gender	Female	1.00	(Reference)			1.00	(Reference)		
	Male	1.47	0.96	2.66	0.08	1.22	0.54	2.78	0.62
Age		1.00	0.98	1.01	0.63	1.01	0.98	1.04	0.69
Duration of symptoms		1.00	1.00	1.01	0.57	1.00	0.99	1.01	0.73
Previous episodes	<2	1.00	(Reference)			1.00	(Reference)		
	>2	0.69	0.43	1.11	0.13	1.06	0.46	2.47	0.89
Distress*						1.00	(Reference)		
1 site						0.89	0.32	2.47	0.83
2 sites						1.30	0.43	3.92	0.64
>2 sites						7.40	0.76	72.51	0.09
Symptoms below knee	No	1.00	(Reference)			NA			
	Yes	1.06	0.63	1.76	0.83	NA			
Sick leave	No	1.00	(Reference)			1.00	(Reference)		
	Yes	1.73	0.88	3.39	0.11	0.88	0.27	2.85	0.84
Physical disability	No	1.00	(Reference)			1.00	(Reference)		
	yes	1.94	1.13	3.36	**0.02**	3.19	1.09	9.33	**0.03**

^a^ Patients having either, 2 or more pain sites, poor sleep or emotional distress.

In the second subgroup with back-related leg pain, both GPs and PTs were significantly less likely to provide information compared with DCs (OR 0.17 and 0.31, respectively). In this subgroup, physical disability was associated with provision of information (OR 3.19, *p* = 0.03) (Table 5).

## Discussion

**Figure 1.**
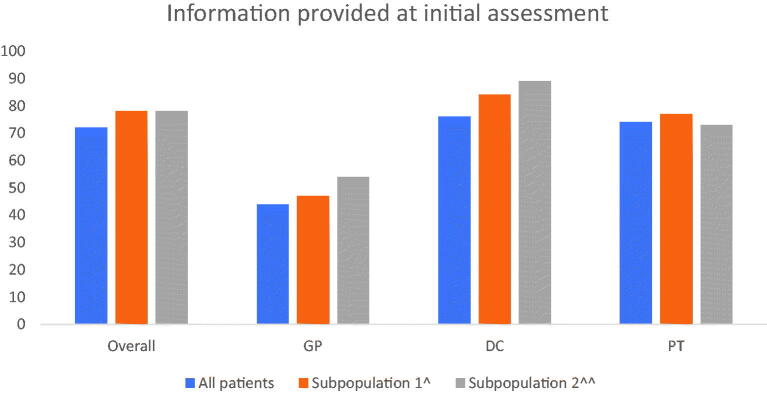
Information at first visit. Proportion of patients who receive information on LBP including prognosis and differences across subpopulations. ^Sub-population 1 is defined as having increased ‘distress’. Distress is defined by having ‘yes’ on one of the following three variables: 2 or more pain sites in addition to LBP, poor sleep, emotional distress. ^^Sub-population 2 is defined by having ‘pain below knee’.

The study aimed to investigate the extent to which GPs, DCs and PTs in Danish primary care provide information to patients with LBP at their first consultation. We found that in 72% of all first-time consultations, the professional registered having provided information on LBP symptoms and prognosis to the patient, but with considerable variation across professions (Figure 1). High-risk sub-populations were more often provided with information at their initial consultation, and elevated distress and physical disability were associated with an increased proportion of patients provided with information.

Even though provision of information is generally recommended in clinical guidelines on LBP [5,6], more than one in four PT and DC patients and more than half of the GP patients did not receive this. This may relate to the recommendations being relatively new and the uptake of the recommendation not complete yet. In one paper of the *Lancet* LBP series from 2018, it was stated that there are gaps between guidelines and daily clinic care, and that focus should be placed on implementing best practice and alignment of practice with evidence [18]. Further, clinical guidelines seldom provide specific and operationalised guidance on what to say to individual patients, which might offer another explanation for the inadequate implementation [19].

This study showed distinct variation across the professions and in the patient populations, which also has been shown earlier [20]. The proportion of patients given information by GPs was significantly lower than by DCs and PTs. The lower provision of information by GPs was observed not only in patients in general, but also in subgroups to which clinic guidelines specifically recommend information on diagnosis and prognosis. In Danish primary care, the tasks of each profession differ. Among the GP’s main tasks are gatekeeping and diagnostics, including referring to relevant primary and secondary health care. In LBP patients, referral has traditionally been mainly to PTs (and to a lesser degree DCs) for treatment. Therefore, it could be speculated that the GP leaves it to the PT (or DC) to provide patient information, and provides information more sparsely. Another explanation could be that most of the patients (62%) have had prior episodes, and GPs may think that sufficient information has already been provided at an earlier stage. This contrasts with DCs and PTs who still provide information in more consultations even though their patients also have had prior episodes. Nonetheless, our findings and suggested explanations are at direct odds with the health information needs of people with LBP: They request general information related to LBP, its cause and underlying pathology, information of general management approaches and self-management strategies – especially in situations with flare-ups [21].

Systemic and organizational procedures, such as time allocated to each patient and referral rights, also differ across the professions, and the differences in allocated time may reflect the differences in provision of information. This is well in line with time restraint being a main clinician barrier to adherence to guidelines [22]. Further, the patient population differs across the three settings, with GPs providing information to fewer patients with LBP compared with PTs and DCs, which may explain the variation in emphasis on providing information across settings.

The study showed that the professionals were overall more likely to give information to patients with elevated distress (widespread pain, poor sleep and emotional distress). Cognitive/psychological distress has been shown to affect prognosis and prolong LBP episodes [9,23], and information on strategies for handling distress has been suggested as a viable means to minimise the risk of a poor outcome. Nevertheless, large differences were seen across professions. In the DC setting, having one of the three distress signs was significantly associated with provision of information, whereas this was only the case in the PT setting if all three signs were present, and non-associated in the GP setting. It has been shown that psychological elements defining the psychosocial profile of patients change across an LBP episode, with high levels of emotional distress in the early stages [24]. This might explain why elevated distress is highly significantly associated with providing information in the DC setting, where patients have significantly shorter duration compared with the GP and PT settings [24]. Differences in patient population may also explain why associations with gender and number of previous episodes are different in the DC setting.

Back-related leg pain has also been described to affect LBP prognosis [11], and the overall level of information provided for this subgroup was higher than for the overall population. Nevertheless, the odds for giving information to this sub-population were significantly lower at PTs and GPs compared with DCs. There are several possible explanations for these differences. First, is the perception of the importance of leg pain in regard to prognosis, and second is the nature of the leg pain (referred or radiating). Further, duration may be a factor in shifting the focus away from back-related leg pain. Patients referred to PTs have a duration of 5 weeks; the prolonged timeframe might explain lower awareness about providing information for this subgroup on prognosis and instead the focus on advice on daily activities. In contrast, an increased focus on leg pain might be a result of the regional reimbursement agreement for DCs introducing a standardised care package for patients presenting with symptoms of lumbar radiculopathy. The structural incentive might increase focus on radiculopathy symptoms, and DCs might allocate more time, be more aware of the representation of leg pain and, therefore, be more vigilant in providing information.

The analyses showed that physical disability was addressed across professions. This is in line with the usual assessment of LBP problems [10] and reflects the expectations of patients who consult for assistance in pain relief and functional improvement [25].

### Strength and limitations

An obvious limitation is the nature of the data. All variables are based on registration from the professionals. Although instructions specified that data entering should be done consecutively, we do not know if data were entered during the consultation, immediately after the consultation or at the end of the day. Further, we do not know how strict the clinicians followed the definitions of single items when entering data. Nevertheless, we know from previous studies using prospective survey registration that data are highly useful and reflect daily clinic activities [15]. In the study, it was recorded whether information was provided or not. We are not able to detect the specific information given or if it was given orally or in writing. Therefore, we are not able to specify the quality or relevance of the given information.

Despite providing clinicians with a common coding manual, the single items in the survey might be understood differently and could therefore affect the data quality. We tried to avoid this by piloting the questionnaire and giving the clinicians the opportunity to consult with the project managers during the project. This resulted in minor rephrasing of paragraphs and adjusting of single items.

A strength of the study is the number of participants across the professions. The large number of consultations allowed us to model the analyses, adjusting for several parameters.

### Perspectives

We have observed substantial differences between the settings as to providing patient information. This may to some extent be explained by inter-professional cultural differences regarding the priority of the various elements of managing patients with LBP, but also by remuneration. It could be argued that future price determination on remuneration and time-slots should better accommodate guideline adherent behaviour by recognising that a full examination, including assessment of important risk factors, and provision of patient information is time consuming.

Regular primary care surveys can be a way to monitor clinical activity and provide information on variations. This could advantageously be combined with interviews to qualify data.

### Conclusion

Across the Danish primary care settings that manage patients with LBP, the guideline-recommended emphasis on patient information is not provided in more than a quarter of first-time consultations. Large variation in providing information exists across the settings, and the proportions provided with information increase for the sub-populations having elevated distress and back-related leg pain below the knee.

## Supplementary Material

Supplemental Material
