# Supplementary material for: Providing information at the initial consultation to patients with low back pain across general practice, chiropractic and physiotherapy – a cross-sectorial study of Danish primary care
Source: Scand J Prim Health Care. 2022 Oct 31;40(3):370–8. doi: 10.1080/02813432.2022.2139465 (PMC9848345; doi:10.1080/02813432.2022.2139465)
Supplement: Supplemental Material [file IPRI_A_2139465_SM2274.docx]

Appendix 1. Overview of the Danish survey charts for general practitioners
